# Plasmon-gating photoluminescence in graphene/GeSi quantum dots hybrid structures

**DOI:** 10.1038/srep17688

**Published:** 2015-12-03

**Authors:** Yulu Chen, Qiong Wu, Yingjie Ma, Tao Liu, Yongliang Fan, Xinju Yang, Zhenyang Zhong, Fei Xu, Jianping Lu, Zuimin Jiang

**Affiliations:** 1 State Key Laboratory of Surface Physics, Key Laboratory of Micro and Nano Photonic Structures (Ministry of Education) and Department of Physics, Fudan University, Shanghai 200433, People’s Republic of China; 2 Department of Physics and Astronomy, University of North Carolina at Chapel Hill, Chapel Hill, NC 27599, USA; 3 SHU-SolarE R&D Lab, Department of Physics, College of Science, Shanghai University, Shanghai 200444, People’s Republic of China

## Abstract

The ability to control light-matter interaction is central to several potential applications in lasing, sensing, and communication. Graphene plasmons provide a way of strongly enhancing the interaction and realizing ultrathin optoelectronic devices. Here, we find that photoluminescence (PL) intensities of the graphene/GeSi quantum dots hybrid structures are saturated and quenched under positive and negative voltages at the excitation of 325 nm, respectively. A mechanism called plasmon-gating effect is proposed to reveal the PL dependence of the hybrid structures on the external electric field. On the contrary, the PL intensities at the excitation of 405 and 795 nm of the hybrid structures are quenched due to the charge transfer by tuning the Fermi level of graphene or the blocking of the excitons recombination by excitons separation effect. The results also provide an evidence for the charge transfer mechanism. The plasmon gating effect on the PL provides a new way to control the optical properties of graphene/QD hybrid structures.

The ability of enhancing light-matter interaction and localizing electromagnetic field of surface plasmons opens up opportunities for light manipulation on low-dimensional structures in a wide range of applications^1^. Graphene would become a new source for electronics and photonics applications due to high Fermi velocity and zero band gap of its carriers^2^. It has been indicated that the graphene sheet can emerge as an alternative two-dimensional plasmonic material that displays a wide range of extraordinary properties^3^,^4^,^5^. Considering the excellent plasmonic characteristics of graphene^6^, people have synthesized different kinds of hybrid structures by using monolayer graphene in contact with a variety of materials, like quantum dots (QDs) and other low-dimensional materials, to explore their optical and electric properties, and photo-responsivity^7^,^8^,^9^,^10^,^11^,^12^,^13^,^14^,^15^,^16^,^17^. Konstantatos *et al*. have exhibited that a graphene/quantum dot hybrid structure has ultrahigh photoresponse gain and high quantum efficiency^7^. Also, Sun *et al*. have reported the ultrahigh infrared photoresponses in a CVD-grown graphene and PbS quantum dots hybrid structure^8^. Koppens *et al*. have reviewed the photodetectors based on graphene, other two-dimensional materials and hybrid systems^12^. A graphene/silicon-heterostructure waveguide photodetector with high-responsivity has been proposed by Wang *et al*.^13^. Further, Engel *et al*. have indicated that the microcavity-induced optical confinement controls the efficiency and spectral selection of photocurrent generation in the integrated graphene device^14^. At the photovoltage field, Echtermeyer *et al*. have obtained the strong plasmonic enhancement of photovoltage in graphene^16^. Bonaccorso *et al*. have reviewed the applications of energy conversion and storage based on the graphene, related two-dimensional crystals, and hybrid systems by considering the plasmonic enhanced absorption^17^. Meanwhile, it has been indicated that the CdSe/ZnS nanocrystals on graphene sheet have a decreased photoluminescence (PL). It is thought that the PL quenching is caused by the electron or energy transferring mechanism^18^. On the contrary, it has been found that graphene/ZnO hybrid structure produces an enhanced photoluminescence for the resonant surface plasmon-polariton (SPP) excitation of graphene in the emission stage^19^. We have also found that the different excited wavelength can modulate the PL enhancement or quenching of graphene/GeSi QDs hybrid structure^20^. In order to obtain the PL enhancement of graphene/semiconductor hybrid structures, the SPP must be effectively excited by incident light.

Huge efforts have been made in effectively controlling the SPP. It has been found that by electric gating and thermal annealing, the dynamic conductivity of graphene device would change largely, and the Fermi energy of graphene is effectively tuned. As a result, the guided light can be modulated within a broad operation spectrum^21^,^22^,^23^. Moreover, by applying a gate voltage, the excitation frequencies of SPP propagating through monolayer graphene can be modulated^24^. For the controlling SPP excitation and the easy tuning Fermi level with external electric fields, the hybrid graphene/QD devices have also attracted an increasing interest^7^,^8^,^25^. For example, in the graphene/PbS QDs, the Fermi energy of graphene is modulated by a voltage applied to the back gate^26^. As a result, the carrier transfer can be controlled by the strength of the built-in field depending on the Fermi level in the graphene. It has been indicated that the photoresponse of the graphene hybrid structure can be tuned in both magnitude and sign with a voltage applied to the back gate of the devices.

Thus, we naturally consider whether the SPP enhanced PL properties of the graphene/GeSi QDs hybrid structure can be controlled by introducing an external electric field. It is also easily considered whether the PL quenching, based on the carrier transfer or the energy transfer of excitons, can be directly indicated by tuning the graphene Fermi level with a bias voltage. The controlling PL properties of QDs are very essential to its application in improving optical properties. In this work, we fabricate the graphene/GeSi QDs hybrid structure devices to explore their optical properties by adding external electric field. It is indicated that the vertical electric field nonlinearly determines the enhancement or quenching of PL intensity at the different excitation wavelengths. At the excitation of 325 nm laser line, which is the resonant excitation wavelength of graphene plasmon^20^, the plasmonic enhanced PL intensity can be modulated by changing the external electric field. With positive voltages, the peculiar PL increasing and then saturating characteristics are explained by a plasmon-gating mechanism, in which the graphene SPP with external electric field hinders the electrons to be transferred away from the surface of multilayer GeSi QDs structure and increases the number of excitons for the recombination. On the contrary, at the excitations of 405 and 795 nm, the PL intensity would decrease with the increasing electric field at positive voltages. It can be explained that the excitons are separated by external electric field. The electrons would drop to under-layer of the hybrid structures, while holes are blocked in the upper-layer GeSi QDs. As a result, the recombination was obstructed, leading to a quenching PL intensity. For all three excitation wavelengths, at the negative voltages, the PL intensity is decreased. The reason is that the negative voltages tune down the Fermi level of graphene, and the graphene sheet as a metal transfers the photogenerated electrons away from GeSi QDs, in similar to ref. 26. The results also directly prove the existence of electron transfer mechanism in the hybrid structure. The PL results indicate that the graphene plasmon can be used to control the luminescence properties of semiconductor by the external vertical field.

## Results

### Morphology and structure characterizations

**Figure 1:**
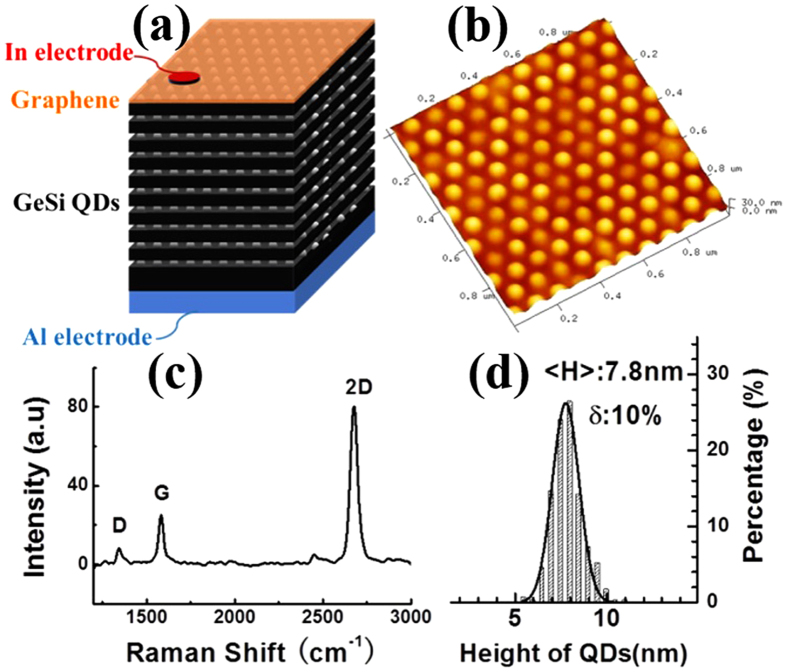
Surface characterizations of hybrid structure device. (**a**) Schematic diagram of the hybrid structure device. (**b**) AFM image (1 μm × 1 μm) of the ten-layer GeSi QDs sample. (**c**) Raman spectrum of the graphene/ten-layer GeSi QDs hybrid structure. D-band represents the defects, G-band means the in-plane vibration of sp^2^ carbon atoms, and 2D-band is for the stacking orders. (**d**) Statistical height distribution of the top layer GeSi QDs.

The schematic diagram of the graphene/ten-layer GeSi QDs hybrid structure device demonstrates that the positive voltage was added on the Al back electrode, and the In point electrode was connected with graphene on the top of the device (Fig. 1a). Figure 1b shows the AFM surface morphology of the graphene/ten-layer GeSi QDs hybrid structure. The average size and height of dots are about 100 and 7 nm (Fig. 1d). The details about the fabrication of the GeSi QDs can be seen in our previous publication^27^. The Raman spectrum of the graphene on GeSi QDs is shown in Fig. 1c. The intensity ratio of 2D-band (~2676 cm^−1^) to G-band (~1580 cm^−1^) is larger than two. The 2D-band is quite shape and cannot be decomposed into two or more components. The characteristics indicate that the graphene on the GeSi QDs is monolayer one. And the low intensity of D-band (1341 cm^−1^) demonstrates the good quality of graphene sheet^28^,^29^.

### Photoluminescence spectroscopic characterizations

**Figure 2:**
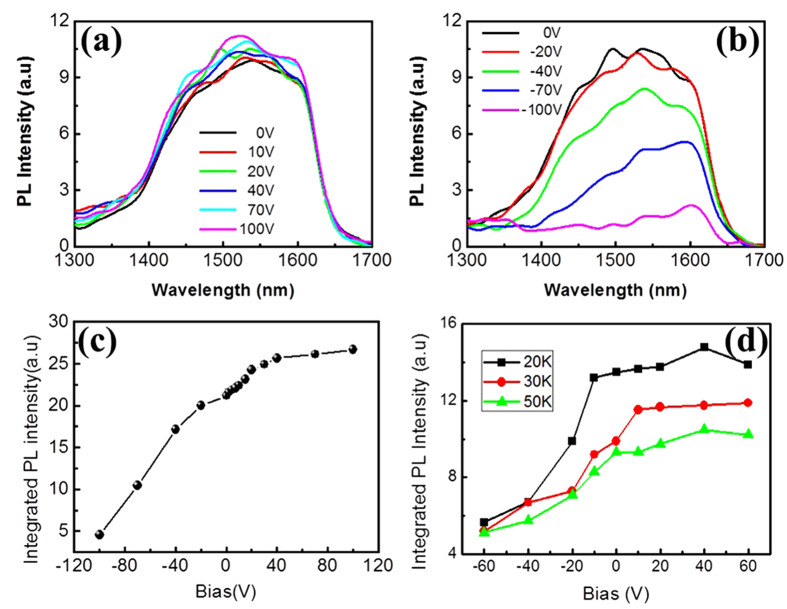
Photoluminescence spectroscopic characterizations of hybrid structure. (**a**) The measured PL spectra of the hybrid structure at the excitation of 325 nm under voltage from 0 to 100 V (added on the back electrode Al). (**b**) The measured PL spectra under voltage from −100 to 0 V. (**c**) The peak-integrated intensities of PL spectra from −100 to 100 V. (**d**) The peak-integrated intensities of PL spectra as a function of temperature at the excitation of 325 nm.

In our previous publication^20^, it has been indicated that the PL intensity of the graphene/GeSi QDs hybrid structure is 1.7 times stronger than that of GeSi QDs without covering the graphene at the excitation of 325 nm. It is called as the SPP enhanced absorption mechanism. Due to the resonant excitation of graphene SPP by incident light, the absorption at the surface region is much enhanced, thus generating more excitons and then increasing the PL intensity in the GeSi QDs. To excite effectively the SPP of graphene by incident light, a modulation periodicity should be introduced to compensate the momentum mismatch, which is suggested to be the intrinsic periodical corrugation of graphene^20^. The corrugation would attenuate or even disappear in the multilayer graphene. The PL enhancement induced by the graphene SPP excited with the incident light may be largely limited for the multilayer graphene or graphite. Here, in this work, we focus on how the SPP enhanced PL spectra of the hybrid structure can be controlled with a bias voltage. First, the PL measurements of the graphene/GeSi QDs hybrid structure were carried out using the excitation of 325 nm laser source with different bias voltages (Fig. 2). The PL peak locates at the wavelength about 1525 nm. The PL intensity slowly increases from 0 to 40 V, and then reaches a saturated value (Fig. 2a). After the voltage reaches 40 V, the integrated PL intensity gradually saturates, and keeps a maximum to 100 V. A new mechanism is proposed to understand the dependence of PL intensity of hybrid structure on the positive voltage, called as plasmon-gating mechanism, as discussed in the later section. On the other hand, the PL intensity decreases largely with decreasing the bias voltages from 0 to −100 V (Fig. 2b). The results indicate that when we add the negative electric field on the hybrid structure, the reduction of Fermi level in graphene to a lower level, like a metal behavior, would cause the electrons in GeSi QDs easily transfer to the surface graphene. Thus, the number of electrons for the recombination is decreased and then the PL intensity quenches. The combinational effect of the SPP enhanced absorption and electron transfer would determine whether the PL intensity increase or not at the exciting wavelength and negative voltage. Figure 2c indicates directly the quenching and enhancement of integrated PL intensity. Also, the PL intensity decreases with the increasing of temperature from 20 to 50 K (Fig. 2d). The result agrees with the general dependence of PL intensity on the temperature of semiconductor materials. The detailed physical mechanism for the reduction and peculiar increasing of the SPP enhanced PL intensity would be discussed in the later section.

**Figure 3:**
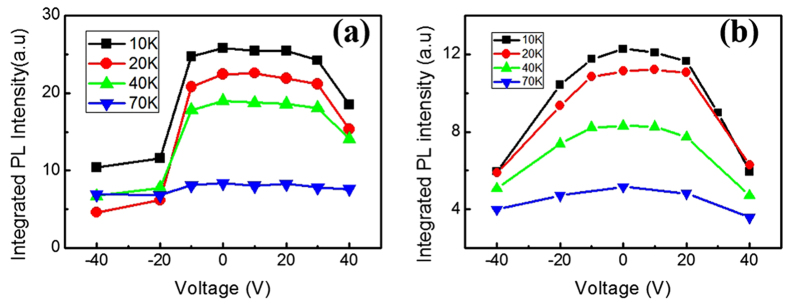
Photoluminescence spectroscopic characterizations of hybrid structure under different wavelength. The measured integrated PL intensities of the hybrid structure at the excitation of 405 nm (**a**), and 795 nm (**b**) under voltage from −40 to 40 V (added on the back electrode Al) as a function of temperature.

Next, the PL spectra of graphene/GeSi QDs hybrid structures are obtained with the other exciting wavelengths for different bias voltages. At the exciting wavelengths of 405 and 795 nm, it is well known that there is no SPP enhanced absorption in graphene^20^. We measure the PL spectra of graphene/GeSi QDs hybrid structures under the biased voltage from −40 to 40 V. It is shown that the integrated PL intensities decrease quickly when the voltage is changed from 0 to −40 V (Fig. 3a,b). The explanation is the same as the discussion in Fig. 2b with negative voltage. It further indicates that electron transfer from GeSi QDs to graphene causes the PL reduction of graphene/GeSi QDs hybrid structures. Under positive voltage, the cases are slightly different between Fig. 3a,b, when we increase the voltage from 0 to 40 V. For the low voltage 0 to 20 V, the PL intensities are almost same and the integrated PL intensities reach a flat roof as an equilibrium status (Fig. 3a). When we further increase the bias voltage, the PL intensity begins to decrease. The quenching PL intensity with increasing bias voltage is observed at different temperatures, as shown in Fig. 3a. When the temperature is at 70 K, the PL signal is quite small so that the intensity change cannot be observed, in according with the general temperature dependence of PL intensity for the semiconductor materials. In Fig. 3b, at the excitation wavelength of 795 nm, the integrated PL intensities are almost symmetrical, both decreasing quickly under the negative and positive bias voltages.

## Discussion

**Figure 4:**
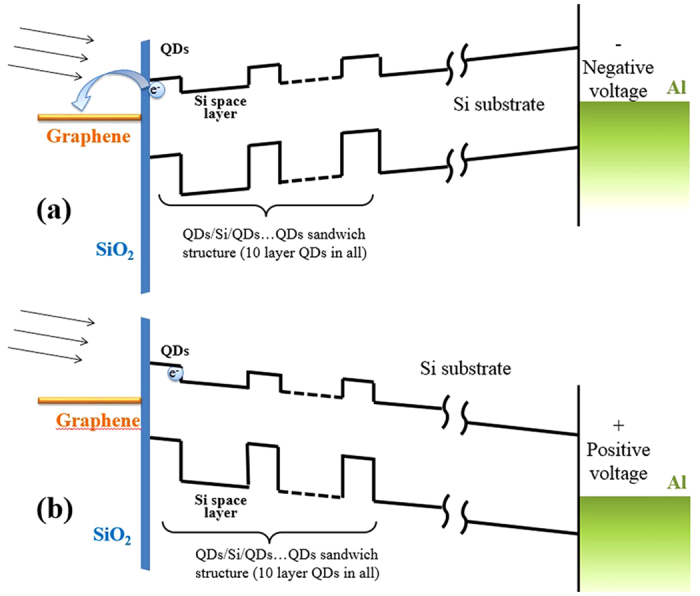
Schematic diagrams of energy band alignment. Incident light (arrows), Fermi level of graphene (orange line), SiO_2_ layer barrier (blue), the energy band alignment of 10 layer GeSi QDs (black), Si substrate, and the Fermi level of Al back electrode (green) are demonstrated respectively under negative voltage (**a**), and positive voltage (**b**).

Figure 4a shows the energy band diagram of the hybrid structure under negative voltage. The PL intensity decreases gradually when the voltage is changed from 0 to −100V in Fig. 2c, from 0 to −40 V in Fig. 3a,b, at the incident light wavelengths of 325, 405 and 795 nm, respectively. This decrement can be attributed to the enhancement of the electron transfer of electron-hole pairs from the GeSi quantum dots to graphene. Under the negative voltages, the oblique potential distribution would induce the excitons separating and produce more electrons in the GeSi QDs. Meanwhile the added electric field reduces the Fermi level of graphene. Thus, the electrons can transfer out the GeSi QDs more easily. The external electric field enhances the electron transfer effect and then causes the reduction of PL intensity. The results also directly prove the existence of electron transfer mechanism in the hybrid structure.

Figure 4b shows the energy band diagram of the hybrid structure under positive voltage. The results seem much complicated. We first discuss the mechanism at the excitation wavelength of 405 and 795 nm laser lines. As shown in Fig. 3a,b, the PL intensity decreases with increasing biased voltage from 0 to 40 V at the excitation of 795 nm. But, at the excitation of 405 nm, the PL intensity reaches a flat roof at around 20 V, and then decreases with increasing voltage. This is because the excitons are separated by the external electric field. The electrons are more likely to drop down to the substrate. As a result, the recombination of electron-hole pairs is limited under large voltage. We notice that the PL intensity has a flat roof within a certain voltage region at 405 nm excitation, as shown in Fig. 3a. Although the surface plasmon of graphene cannot be effectively excited, the wavelength 405 nm is slightly close to 325 nm. Thus, non-resonant SPP excitation may exist. Thus, in a certain voltage range, the SPP non-resonant excitation and excitons separation can reach an equilibrium status. In Fig. 3b, however, the excitation of 795 nm is too far away from the resonant SPP wavelength of graphene, the plasmon cannot be excited by the light. Under an external electric field, the excitons are separated quickly, the recombination of electron-hole pairs is blocked, and then the PL intensity is decreased. Thus, there is the PL quenching at higher voltage.

At the excitation of 325 nm and under positive biased voltage, the results look quite interesting. From Fig. 2c, the integrated PL intensity gradually increases from 0 to 40 V, and finally saturates at the voltage exceeding 40 V. According to our previous publication^20^ as well as the description in the Results section, the SPP of graphene can be excited by 325 nm incident light. The field of SPP perpendicular to the graphene/QD interface decays exponentially and its skin depth is about several nanometers. There is only a 2 to 3 nm thick naturally formed SiO_2_ layer between the first layer GeSi QDs and the graphene. Thus, the SPP can influence the topmost layer GeSi QDs. The resonant excitation of graphene SPP can enhance the absorption at surface region so that the generation of electron-hole pairs in the topmost layer QDs is enhanced. For the hybrid structure, there are two competing mechanisms. One is the SPP absorption mechanism to enhance the PL intensity, and another is the electron transferring from QDs to the graphene, which reduce the PL intensity. Under the positive voltage, the oblique band alignment shown in Fig. 4b can reduce the transferring of electrons. Thus, the PL intensity increases.

Similar to photogating effect in the graphene covered PbS QDs structure, in which the photogenerated charges can transfer to the graphene and the oppositely charged carriers remain trapped in the QD layer under the build-in field at the QD/graphene interface^7^, we named the mechanism for the SPP enhanced PL under positive biased voltage as plasmon-gating effect. The oscillating positive and negative charge regions are induced by SPP in the graphene. The forming oscillating dipoles can cause an added in-plane force on electrons and holes in the QDs. The in-plane force may limit the electrons to be transferred out the plane of top QDs layer and the electron-hole pairs to be separated by the external electric field. Thus, the recombination of the e-h pairs which gives out the luminescence can be kept.

In summary, we have fabricated a graphene/GeSi QDs hybrid structure device and measured the PL properties under varying bias voltages at different excitation wavelengths. At all three exciting wavelengths, the PL intensity of the hybrid structure is found to be quenched with increasing negative biased voltage on the graphene/QDs structure, which can be explained by the electron transfer model. Under positive biased voltage, different dependences of PL intensity of the hybrid structure on the voltages are observed at three excitation wavelengths. At the excitation of 325 nm laser line, a plasmon-gating mechanism is proposed for the observed PL intensity dependence on the positive biased voltage. At the excitations of 405 and 795 nm, the PL intensity of the hybrid structure is found to be quenched with increasing positive bias voltage, which is explained by excitons separation effect. The gating of the plasmon resonance excitation and its effect on the photoluminescence provides a new way to control the optical properties of graphene/QD hybrid structures.

## Methods

### Sample fabrication

10 layers ordered GeSi QDs sample was grown on pit-patterned Si (001) substrates with periods of 100 nm by solid source Molecular Beam Epitaxy. Large-scale graphene layer was fabricated by chemical vapor deposition (CVD) on a Cu substrate. The structure of graphene was confirmed by Fourier transform infrared (FTIR)-Raman spectra with the excitation of 325 nm laser line. Surface morphology of the sample was characterized using atomic force microscopy (AFM). Then the monolayer graphene was transferred on to the surface of GeSi QD sample to form graphene/GeSi QDs hybrid structures with the aid of polymethylmethacrylate (PMMA).

### Device

In order to add the vertical electric field on the hybrid structure, the In and Al contacts are deposited as the front and back electrodes. The back electrode was formed by thermal evaporation of 300 nm Al on the back of the silicon wafer, and the front electrode was formed by alloying In contact at the top of graphene layer with about 4 mm^2^. Two electrical lines from the probes were pressed on the In electrode and the back side. The vertical electric fields can added to the sample in the vacuum chamber using an external 2400 Keithley Source Meter. We provide stable applied bias voltage from −100 V to 100 V.

### PL measurement

The PL measurements under different voltages were performed in a He-flow cryostat with the 325 nm line of HeCd laser, and 405 and 795 nm lines of diode lasers as the excitation source. The spot size of laser can be focused to 1 mm^2^. The PL spectra were recorded by an extended InGaAs detector using the standard lock-in technique.

## Additional Information

**How to cite this article**: Chen, Y. *et al*. Plasmon-gating photoluminescence in graphene/GeSi quantum dots hybrid structures. *Sci. Rep.* **5**, 17688; doi: 10.1038/srep17688 (2015).

## References

[b1] Clavero C. Plasmon-induced hot-electron generation at nanoparticle/metal-oxide interfaces for photovoltaic and photocatalytic devices. Nature Photon . 8, 95 (2014).

[b2] Geim A. K. & Novoselov K. S. The rise of graphene. Nature Mater . 6, 183 (2007). 17330084 10.1038/nmat1849

[b3] Wang B., Zhang X., Garcia-Vidal F., Yuan X. & Teng J. Strong Coupling of Surface Plasmon Polaritons in Monolayer Graphene Sheet Arrays. Phys. Rev. Lett. 109, 073901 (2012). 23006371 10.1103/PhysRevLett.109.073901

[b4] Grigorenko A. N., Polini M. & Novoselov K. S. Graphene plasmonics. Nature Photonics 6, 749 (2012).

[b5] Thongrattanasiri S. & Javier Garcia de Abajo F. Optical Field Enhancement by Strong Plasmon Interaction in Graphene Nanostructures. *Phys. Rev. Lett*. 110, 187401 (2013). 23683241 10.1103/PhysRevLett.110.187401

[b6] Fei Z. . Gate-tuning of graphene plasmons revealed by infrared nano-imaging. Nature 487, 82 (2012). 22722866 10.1038/nature11253

[b7] Konstantatos G. . Hybrid graphene–quantum dot phototransistors with ultrahigh gain. Nature Nanotech . 7, 363 (2012). 10.1038/nnano.2012.60 22562036

[b8] Sun Z. . Infrared photodetectors based on CVD-grown graphene and PbS quantum dots with ultrahigh responsivity. *Adv. Mater*. 24, 5878 (2012). 22936561 10.1002/adma.201202220

[b9] Konstantatos G. . Ultrasensitive solution-cast quantum dot photodetectors. Nature 442, 180 (2006). 16838017 10.1038/nature04855

[b10] Böberl M., Kovalenko M. V., Gamerith S., List E. J. W. & Heiss W. Inkjet-Printed Nanocrystal Photodetectors Operating up to 3μm Wavelengths. *Adv. Mater*. 19, 3574 (2007).

[b11] Fang Z. . Plasmon-Induced Doping of Graphene. *ACS Nano*. 6, 10222 (2012). 22998468 10.1021/nn304028b

[b12] Koppens F. H. L. . Photodetectors based on graphene, other two-dimensional materials and hybrid systems. Nature Nanotech . 9, 780 (2014). 10.1038/nnano.2014.215 25286273

[b13] Wang X. M., Cheng Z. Z., Xu K., Tsang H. K. & Xu J. B. High-responsivity graphene/silicon-heterostructure waveguide photodetectors. Nature Photon . 7, 888 (2013).

[b14] Engel M. . Light–matter interaction in a microcavity-controlled graphene transistor. Nature Commun . 3, 906 (2012). 22713748 10.1038/ncomms1911 PMC3621428

[b15] Furchi M. . Microcavity-Integrated Graphene Photodetector. *Nano Lett*. 12, 2773 (2012). 22563791 10.1021/nl204512x PMC3396125

[b16] Echtermeyer T. J. . Strong plasmonic enhancement of photovoltage in graphene. Nature Commun . 2, 458 (2011). 21878912 10.1038/ncomms1464

[b17] Bonaccorso F. . Graphene, related two-dimensional crystals, and hybrid systems for energy conversion and storage. *Science*. 347, 1246501 (2015). 25554791 10.1126/science.1246501

[b18] Chen Z., Berciaud S., Nuckolls C., Heinz T. F. & Brus L. E. Energy Transfer from Individual Semiconductor Nanocrystals to Graphene. *ACS Nano*. 4, 2964 (2010). 20402475 10.1021/nn1005107

[b19] Hwang S. W. . Plasmon-Enhanced Ultraviolet Photoluminescence from Hybrid Structures of Graphene/ZnO Films. *Phys. Rev. Lett*. 105, 127403 (2010). 20867671 10.1103/PhysRevLett.105.127403

[b20] Chen Y. L. . Effect of graphene on photoluminescence properties of graphene/GeSi quantum dot hybrid structures. *Appl. Phys. Lett*. 105, 021104 (2014).

[b21] Horng J. . Drude conductivity of Dirac fermions in graphene. *Phys. Rev. B*. 83, 165113 (2011).

[b22] Ren L. . Terahertz and Infrared Spectroscopy of Gated Large-Area Graphene. *Nano. Lett*. 12, 3711 (2012). 22663563 10.1021/nl301496r

[b23] Liu M. A graphene-based broadband optical modulator. *Nature*. 474, 64 (2011). 21552277 10.1038/nature10067

[b24] Gao W. . Excitation and Active Control of Propagating Surface Plasmon Polaritons in Graphene. *Nano Lett*. 13, 3698 (2013). 23895501 10.1021/nl401591k

[b25] Zhang D. Y. . Understanding Charge Transfer at PbS-Decorated Graphene Surfaces toward a Tunable Photosensor. *Adv. Mater*. 24, 2715 (2012). 22505476 10.1002/adma.201104597

[b26] Huang Y. Q., Zhu R. J., Kang N., Du J. & Xu H. Q. Photoelectrical response of hybrid graphene-PbS quantum dot devices. *Appl. Phys. Lett*. 103, 143119 (2013).

[b27] Ma Y. J. . Formation of coupled three-dimensional GeSi quantum dot crystals. *Appl. Phys. Lett*. 100, 153113 (2012).

[b28] Li X. . Large-Area Graphene Single Crystals Grown by Low-Pressure Chemical Vapor Deposition of Methane on Copper. J. Am. Chem. Soc. 133, 2816 (2011). 10.1021/ja109793s 21309560

[b29] Ferrari A. C. . Raman Spectrum of Graphene and Graphene Layers. *Phys. Rev. Lett*. 97, 187401 (2006). 17155573 10.1103/PhysRevLett.97.187401

